# Alcohol Drinking Cessation and the Risk of Laryngeal and Pharyngeal Cancers: A Systematic Review and Meta-Analysis

**DOI:** 10.1371/journal.pone.0058158

**Published:** 2013-03-01

**Authors:** Aliasghar Ahmad Kiadaliri, Johan Jarl, Georgios Gavriilidis, Ulf-G Gerdtham

**Affiliations:** 1 Division of Health Economics, Department of Clinical Sciences, Malmö University Hospital, Lund University, Malmö, Sweden; 2 Department of Health Management and Economics, School of Public Health, Tehran University of Medical Sciences, Tehran, Iran; 3 Health Economics & Management, Institute of Economic Research, Lund University, Lund, Sweden; 4 Department of Economics, Lund University, Lund, Sweden; Bremen Institute of Preventive Research and Social Medicine, Germany

## Abstract

**Objective:**

To evaluate the effect of alcohol cessation on the risk of developing laryngeal and pharyngeal cancers, combining available evidence in the scientific literature in a meta-analysis.

**Methods:**

A systematic literature review was conducted, and a meta-analysis was applied on the retrieved studies. The generalised least squares method was used to estimate the trend from dose-response data to assess changes in the risks of laryngeal and pharyngeal cancers after drinking cessation.

**Results:**

A total of 9 case-control studies were included in the meta-analysis (4 and 8 estimates for laryngeal and pharyngeal cancers, respectively). On average, alcohol drinking cessation was associated with a 2% yearly reduction in the risk of developing laryngeal and pharyngeal cancers. There was a considerable heterogeneity between the studies of pharyngeal cancer, but this was mostly due to two studies. The increased risk of laryngeal and pharyngeal cancers caused by alcohol was reversible; the time periods until the risks became equal to those of never drinkers were 36 (95% CI 11–106) and 39 (95% CI 13–103) years, respectively. Moreover, 5 years of drinking cessation was associated with a reduction of around 15% in the alcohol-related elevated risk of laryngeal and pharyngeal cancers.

**Conclusion:**

Although a long time period is required to completely eliminate the alcohol-related elevated risk of laryngeal and pharyngeal cancers, a substantial risk reduction can be seen in the short term (5–10 years), and drinking cessation should therefore be encouraged to reduce the incidence of these cancers.

## Introduction

Oral cavity, oropharynx, hypopharynx, and larynx cancers, when grouped together as head and neck cancer, constitute the seventh most common form of cancer in the world [1]. In 2008, an estimated 482,000 new cases of lip, oral cavity, nasopharynx, and other pharynx cancers were diagnosed worldwide. For laryngeal cancer, the corresponding figure was 151,000. In the same year, these cancers were responsible for 355,000 deaths worldwide [2]. This implies that significant health benefits could be achieved via effective prevention and treatment strategies.

Several studies have shown that alcohol consumption increases the risk of cancer of the oral cavity, pharynx, and larynx [3]–[5]. For example, Baan et al. [6] found that drinking 50 g of pure alcohol per day was associated with a 2–3 times higher risk of developing these cancers compared with non-drinkers. Cessation or reduction of alcohol drinking could therefore be an effective strategy for reducing the risk of diseases. However, there is a lack of consistency in the literature regarding the impact of alcohol cessation on the risk of head and neck cancer [7]. In other words, it is not clear to what extent the increased risk of head and neck cancer due to alcohol consumption is reversible through cessation, nor it is clear how fast this risk may decline. If the elevated risk of alcohol consumption is not reversible, then the focus of prevention programs should be on preventing and/or delaying drinking initiation, while if the elevated disease risk is reversible, cessation programmes may be a cost-effective option to fight these types of cancers in high-risk groups.

A previous study-level meta-analysis by Rehm et al. [8] examined the association between alcohol cessation and the risk of head and neck cancer and reported the risk for some time points after drinking cessation. They found that the risk of head and neck cancer elevated up to 10 first years of quitting drinking and reduced after this point.

Separate analysis of different types of head and neck cancer may result in different trends compared to the results from pooled analysis [10]. Hence, two separate analyses were conducted in order to answer the following research questions: What is the existing knowledge about the effect of drinking cessation on the risk of laryngeal and pharyngeal cancers? Specifically, is the risk reversible and, if so, how fast does it fall? Therefore a meta-analysis was applied based on relevant studies identified through a systematic literature review and appropriate estimation methods.

## Materials and Methods

### Search strategy

A systematic literature review was performed in July 2010 by one author (GG) and independently verified and updated in February–March 2012 and December 2012 by another author (AAK). PubMed/MEDLINE, EMBASE, SCOPUS, OVID and Web of Science were searched using the following terms: [“alcohol” AND (“laryngeal” OR “larynx” OR “upper aerodigestive tract” OR “head and neck” OR “oral” OR “oropharyngeal” OR “pharyngeal”) AND “risk” AND (“cancer OR carcinoma*” OR “neoplasm” OR “neoplastic” OR “squamous cell”) AND (“cessation” OR “abstinence” OR “abstainers” OR “quit drinking” OR “quitting drinking” OR “stop drinking” OR “stopping drinking”)]. The PRISMA guidelines [11] were followed in this process.

### Selection of studies

Five exclusion criteria were applied. A study was excluded from the review if: 1) it did not investigate laryngeal or pharyngeal cancer (ICD-10 codes CO1–C10; C12–C14; C32; DOO; and DO2.0); 2) it was not published in English; 3) it did not quantitatively capture the effect of time since drinking cessation on the risk of laryngeal or pharyngeal cancer; 4) it was a review article; or 5) it was not conducted on humans. Studies that included individuals who already had the disease (e.g. those studying the effect of drinking cessation on recovery) were also excluded.

**Figure 1:**
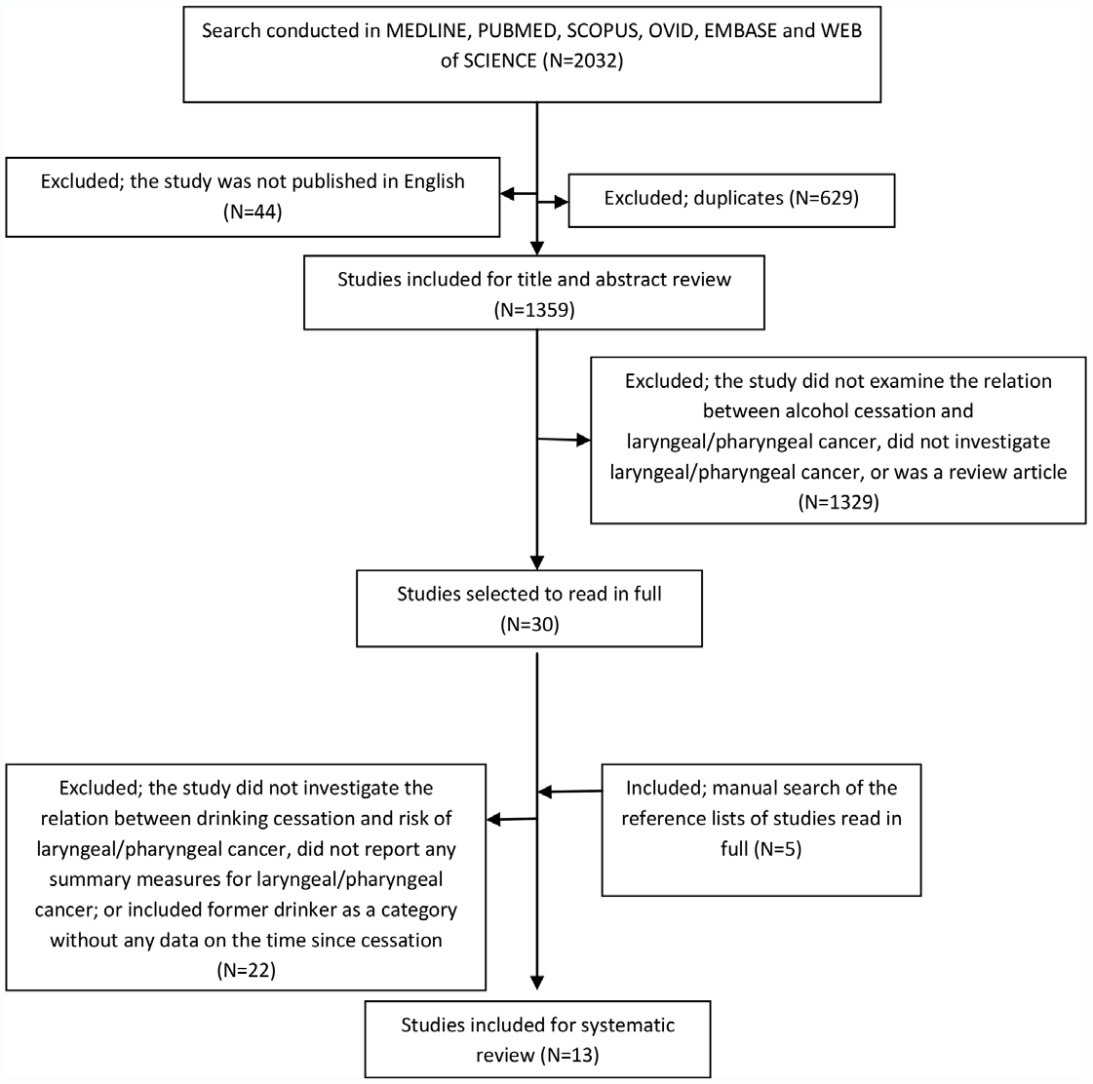
Flow diagram of literature search.

The initial search resulted in 2032 articles. After excluding the duplicates and non-relevant studies, 30 articles were selected for full text examination. The reference lists of these 30 studies were searched manually. In total, 13 articles passed the exclusion criteria for the systematic review. Figure 1 shows the process of study selection. Among these 13 studies, the study by Takezaki et al. [12] did not present sufficient data for meta-analysis and was excluded from that part of study. In addition, the study by Rehm et al. [8] was a meta-analysis including some of the other studies identified in our search, and was therefore excluded from our meta-analysis. As, the study by Marron et al. [7] included the studies by Franceschi et al. [24] and Hayes et al. [25], these two studies were not included in our meta-analysis to avoid giving too much weight to these specific samples. The study by Marron et al [7] was an individual-level analysis and the results of most of the studies included in their analysis were not published elsewhere. In terms of the site of cancer, four studies reported the risk estimates for laryngeal cancer and ten for pharyngeal cancer. It should be noted that some studies reported the risk estimates for both types of cancers, and so a total of 12 estimations from 9 studies were used in the meta-analysis.

### Data extraction

The main data extracted from the selected studies included: time since drinking cessation and related risk estimates with confidence intervals, type of study, country, study years, sample size, age and gender characteristics of sample, and statistical method. An additional piece of information collected from the included studies was the definition of former drinker. In order to avoid the ‘sick quitter’ effect (i.e. individuals quitting drinking due to the disease), we made a distinction between studies that required a time lag between drinking cessation and being defined as a former drinker and studies that did not require a time lag. The reason for this is that comparing healthy drinkers with sick quitters who have higher risk of developing laryngeal and pharyngeal cancers may underestimate the efficacy of cessation [13]. As years since drinking cessation were reported in categories, the midpoints of the categories were used as a dose measure. Where categories were open-ended, the same interval width as the previous category was applied. Current drinkers were used as the reference category in the analysis. For studies using a different reference group, we recalculated the risk estimates and their confidence intervals using the method proposed by Hamling et al. [14]. In this method, first a table including the number of cases and controls in each category is constructed and then these numbers are grouped together in a 2×2 table by exposures and then ORs and CIs are calculated using relevant equations (for detailed information see [14]).

### Statistical analysis

The effect measure of interest was relative risk (RR), but as all retrieved studies were case-control, they reported odds ratios (ORs). When the prevalence of a disease is low, these two values are approximately equal [15]. Mathematically-speaking, it is easier to use ORs in meta-analysis [16], [17], and so we treated all measures as ORs while interpreting the results as relative risks.

To model the dose-response relationship between years since drinking cessation and the risk of disease, we used the method proposed by Greenland et al. [18] and developed by Orsini et al. [9]. In this method, the generalized least squares (GLS) technique is used to estimate the β vector of regression coefficients in the following model [9]:
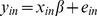
where y is an n×1 vector of reported odds ratios, x is an n×k matrix of k covariates for study i and dose level n, β is a k×1 vector of regression coefficients, and e is an n×1 vector of random errors. The main advantage of this method is that it allowed us to account for the correlation among the risk estimates across alcohol cessation categories, and hence to avoid the underestimation caused by traditional methods [9].

The risks of laryngeal and pharyngeal cancers due to a one-year increase in the duration of alcohol cessation were calculated based on the pooled data. Statistical heterogeneity among studies was examined using the Q statistic (P-value <0.05) [19]. If any heterogeneity was present, effect modifiers based on the characteristics of the included studies were added to the model to explain the heterogeneity. In this case, the coefficients on these effect modifiers show the dose-response relation in a subgroup of the studies. If heterogeneity remained, a random-effects model was applied. This model assumes that the studies estimate different underlying effect sizes, and incorporates this between-study variation into the analysis [20]. Separate models were developed for laryngeal and pharyngeal cancers. To examine any nonlinearity in the log-linear dose-response relations, we used the cubic splines method; this allowed us to assess nonlinearity both graphically and by a formal statistical test [21]. In this model, a four-knot restricted cubic spline transformation was applied to the pooled dose data, and the joint null hypothesis that the regression coefficients of the spline transformations are all equal to zero was tested [21]. Contour-enhanced funnel plots [22] and the Egger’s regression asymmetry test [23] (p<0.05 was considered representative of statistically significant publication bias) were used to check for any potential publication bias in our meta-analysis.

To check the influence of each study on the results, a sensitivity analysis was performed omitting each study in turn and then re-estimating the summary effect of remaining studies. An additional sensitivity analysis was performed for pharyngeal cancer, due to the fact that the studies by Balaram et al. [38]and Garrote et al. [41] did not report that cases were histologically confirmed. All analyses were performed using version 11 of the Stata software package [26].

To quantify the length of time taken after drinking cessation for the alcohol-related elevated risk of laryngeal and pharyngeal cancers to fall to the level of never drinkers, another meta-analysis was conducted. In this analysis, the risk of never drinkers compared with current drinkers as reported in the included studies was estimated using Forrest plots. This estimate was combined with the estimated yearly risk reduction due to drinking cessation to quantify the number of years required until the elevated risk of alcohol drinking disappears.

## Results

The characteristics of the 13 studies identified in the systematic literature review are shown in Table 1 (more details on the characteristics of the studies are given in Table S1 in the supplemental). The studies were published between 1969 and 2011, and covered years ranging from 1966 to 2000. Five were conducted in Latin America and the Caribbean region, three in Europe, and three in Asia, while one was a pooled analysis of studies mainly conducted in the USA. Eleven of the studies had a case-control design, and one was a pooled analysis of case-control studies [7]. The cancer cases were histologically confirmed in most studies, and the hospital was the main source of controls in the majority of the included studies. The age of the participants ranged from 15 years to over 90. Gender-specific estimates were calculated in four studies, although three of them focused only on men. In all studies, men constituted the main proportion of the cases, and all studies but two [12], [27] applied matching of cases and controls mainly on age and gender (see Table S1 in the supplemental). The study by Martinez et al. [28] was the only one which did not control for any confounder in calculating the ORs and did not require any time lag between quitting drinking and being defined as a former drinker.

**Table 1:** Characteristics of studies of the risk of laryngeal and pharyngeal cancers following drinking cessation.

Study	Country and study years	Type of study	Study area	Mortality/morbidity	Age	Gender specific calculations	Gender cases (% male)
Altieri et al., 2002 [37]	Italy and Switzerland, 1992–2000	Case-control	Larynx	Morbidity	30–79	No	91
Balaram et al., 2002 [38]	India, 1996–1999	Case-control	Oral cavity	Morbidity	20–85	Yes (men only)	100
Castellsague et al., 2004 [39]	Spain, 1996–1999	Case-control	Oral cancer	Morbidity	< = 51–> = 70	No	81
De Stefani et al., 2004 [40]	Uruguay, 1997–2003	Case-control	Hypopharyngeal, larynx	Morbidity	30–89	Yes (men only)	100
Franceschi et al., 2000 [24] 1	Italy and Switzerland, 1992–1997	Case-control	Oral cavity, pharynx	Morbidity	<40–79	No	85
Garrote et al., 2001 [41]	Cuba, 1996–1999	Case-control	Oral cavity, oro-pharynx	Morbidity	25–91	No	72
Hayes et al., 1999 [25] 1	Puerto Rico, 1992–1995	Case-control	Oral cavity, pharynx, salivary gland	Morbidity	21–79	Yes	81
Marron et al., 2009 [7]	–	Pooledcase-control	Oropharynx, hypopharynx, larynx,oral cavity	Morbidity	15–80	No	NA
Martinez, 1969 [28]	Puerto Rico, 1966	Case-control	Pharynx, oral cavity	Morbidity	NA	No	87
Rehm et al.^1^, 2007 [8]	–	Meta-analysis	Head and neck cancer	See included	See included	See included studies	See included
Szymanska et al., 2011 [42]	Brazil, Argentina and Cuba, 1998	Case-control	Oral cavity, oro-pharynx,hypopharynx, larynx	Morbidity	<40–>80	No	85
Takezaki et al., 1996 [27]	Japan (Aichi), 1988–1993	Case-control	Tongue, mouth, oropharynx, hypopharynx	Morbidity	20–79	No	71
Takezaki et al., 2000 [12] 1	Japan (Nagoya), 1988–1997	Case-control	Hypopharynx	Morbidity	40–79	Yes (men only)	100

1 These studies were not included in the meta-analysis part of the current study.

### Laryngeal cancer

**Figure 2:**
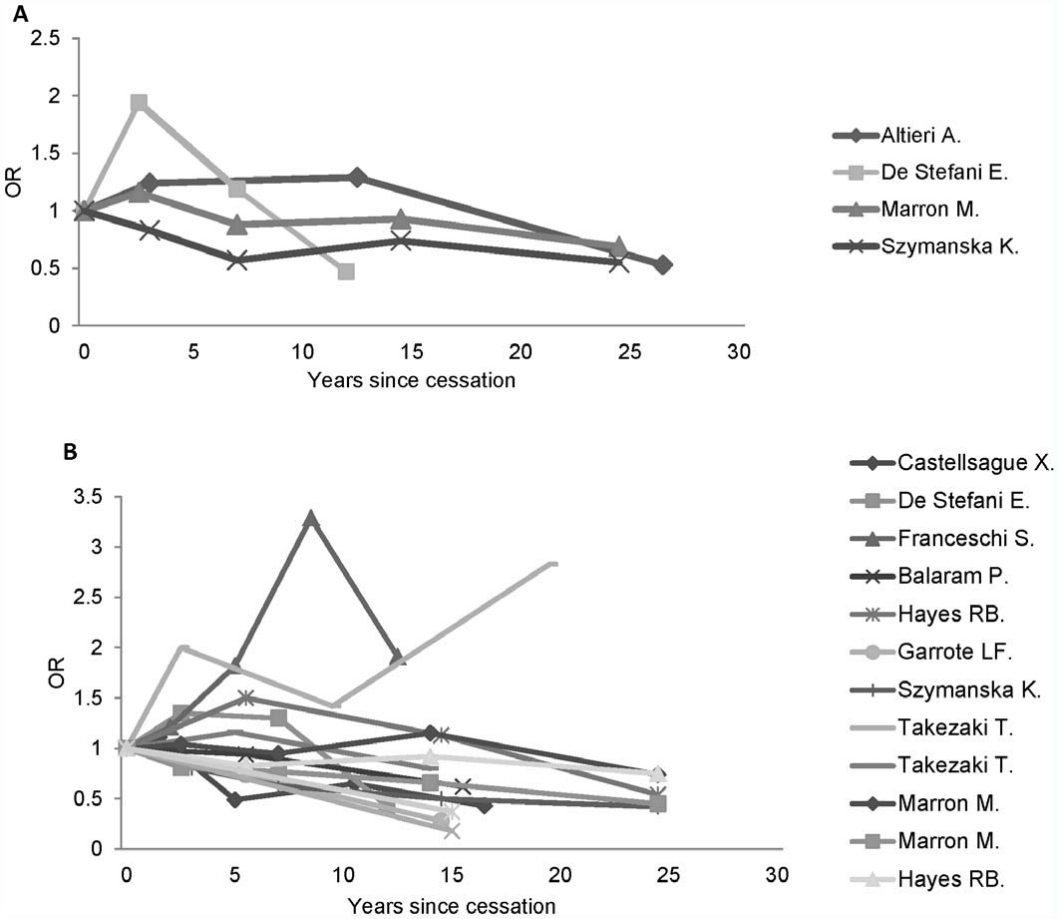
Risk of developing laryngeal cancer and pharyngeal cancer following drinking cessation.

Figure 2A shows the relationship between years since drinking cessation and risk of laryngeal cancer as reported in the included studies. In general, the studies showed an increasing risk over the initial years after quitting, followed by a decreasing trend.

Table 2 shows the results of meta-regression analysis. None of the effect modifiers were significant, and so Model 1 was the preferred model. The risk of developing laryngeal cancer fell by 2% on average per year of cessation, so, for example a person who quit drinking alcohol ten years ago would have 82% of the risk of a current drinker. There was no evidence of a nonlinear relationship between the cessation of alcohol drinking and the risk of laryngeal cancer (P for nonlinearity = 0.98; Figure S1 in the supplemental).

**Table 2:** Meta-analysis of changes in the odds ratios of laryngeal and pharyngeal cancers after drinking cessation compared to current drinkers.

Variables	Model 1	Model 2	Model 3	Model 4	Model 5	Model 6
**Laryngeal**						
Additional year of drinking cessation (dose)	0.98***	0.98***				
Dose*study conducted in Europe		1.02				
Q statistics†	18.42	17.58				
**Pharyngeal**						
Additional year of drinking cessation (dose)	0.98***	0.98***	0.98***	0.98***	0.98***	0.98***
Dose*study conducted in Europe		0.97				0.97
Dose*study did not control for smoking			0.95**		0.95***	0.95**
Dose*no matching in the study				1.08***	1.08***	1.08***
Q statistic†	52.91***	50.79***	47.26***	43.97**	38.70**	36.59**

*** ,**,*show 1%, 5%, and 10% significant levels, respectively.

† H0: No heterogeneity.

**Figure 3:**
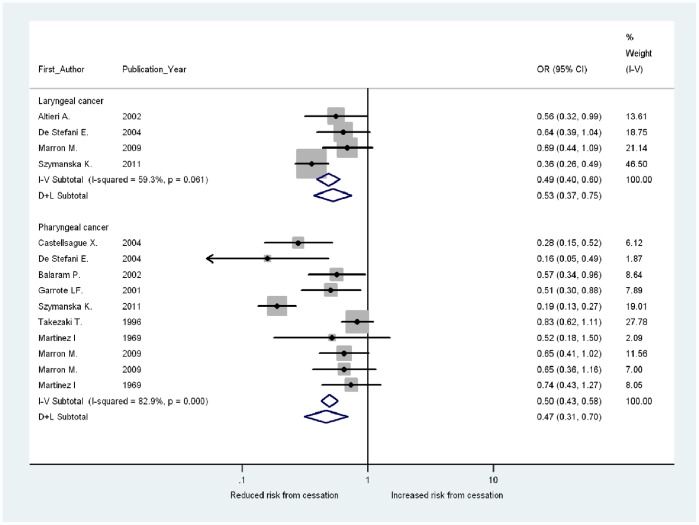
Risk of developing laryngeal and pharyngeal cancer for never drinkers vs. current drinkers.

**Figure 4:**
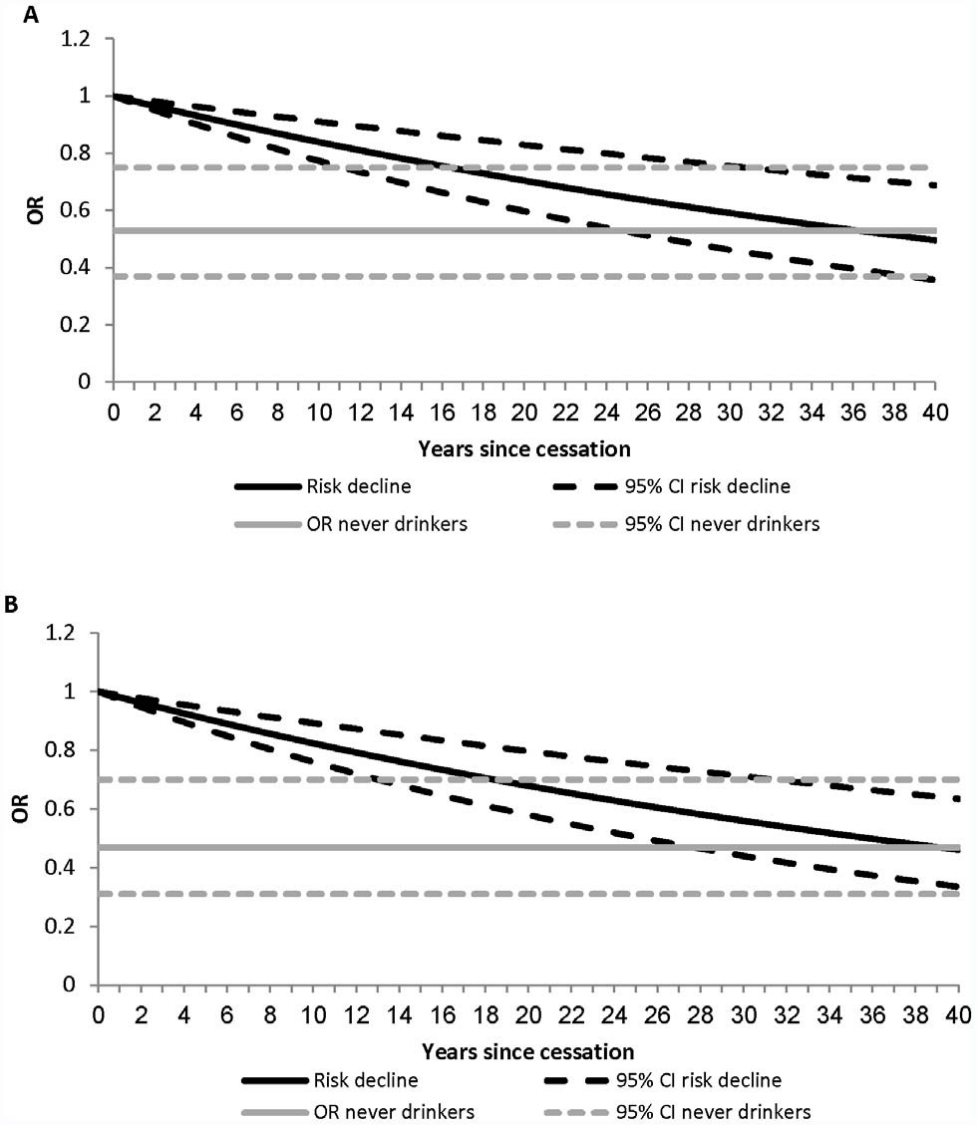
Risk decline of laryngeal and pharyngeal cancer over forty years after drinking cessation. It should be noted that the risk is not expected to fall below that of never drinkers, even though the figures imply this.

The risk of developing laryngeal cancer was 47% (OR 0.53, 95% CI 0.37–0.75) lower for never drinkers than for current drinkers (Figure 3). Combining this figure with the results of the meta-regression implies that the alcohol-related elevated risk of laryngeal cancer would last 36 (95% CI: 11–106) years after drinking cessation (Figure 4A).

### Pharyngeal cancer

The associations between years since drinking cessation and risk of pharyngeal cancer in the included studies are shown in Figure 2A, and the results of the meta-regression analysis in Table 2. There was considerable heterogeneity between the studies of pharyngeal cancer, which could not be fully explained by controlling for observable factors. Hence, random-effects models were used to capture the unexplained heterogeneity between studies. Based on the Q statistic and effect modifiers significance level, Model 5 was considered the preferred model. The results of this model revealed that alcohol cessation reduces the risk by 2% per year on average, which corresponds to an 18% fall in the risk of pharyngeal cancer after 10 years compared with current drinkers. This result was based on the studies which controlled for smoking and used matching in the study (6 out of 8 studies). The fall in risk was markedly higher in the study that did not control for smoking [28], while the risk actually increased over time in the non-matched case-control study [27]. The cubic spline analysis showed no evidence of a nonlinear association between drinking cessation and the log-risk of pharyngeal cancer (P for nonlinearity = 0.46; Figure S2 in the supplemental). The risk of developing pharyngeal cancer was 53% (OR 0.47; 95% CI 0.31–0.70) lower for never drinkers compared with current drinkers (Figure 3). This implies that the alcohol-related elevated risk of pharyngeal cancer would last 39 (95% CI 13–103) years after drinking cessation (Figure 4B).

### Sensitivity analysis

As shown in Table 3, excluding the studies by Balaram et al. [38] and Garrote et al. [41] in the meta-analysis did not change the OR of one additional year of drinking cessation. In other word, there was no significant difference in the risk between studies which confirmed the cancer cases histologically and those which did not [38], [41]. In this case, the risk of developing pharyngeal cancer was 55% (OR 0.45; CI: 0.27–0.74) lower for never drinkers compared with current drinkers, meaning that the alcohol-related elevated risk of pharyngeal cancer would last 39 (95% CI: 11–103) years.

**Table 3:** Sensitivity analysis of the meta-analysis of changes in the odds ratio of pharyngeal cancer after drinking cessation compared to current drinkers.

Variables	Model 1	Model 2	Model 3	Model 4	Model 5	Model 6
Additional year of drinking cessation (dose)	0.98***	0.98***	0.98***	0.98***	0.98***	0.98***
Dose*study conducted in Europe		0.97			0.	0.97
Dose*study did not control for smoking			0.95**		0.95**	0.95**
Dose*no matching in the study				1.08***	1.08***	1.08***
Q statistic^†^	49.48***	47.25***	43.65***	40.73***	35.28**	33.06**

See footnote in Table 2. Excluding studies 38 & 41.

The results of another sensitivity analysis showed that no single study had a significant impact on the results. In addition, the results of contour-enhanced funnel plots showed that there was not enough evidence to conclude that the results suffered from publication bias (Figures S3 and S4 in the supplemental). The Egger’s test also confirmed the lack of strong evidence for publication bias in our meta-analysis (P = 0.404 for laryngeal and P = 0.339 for pharyngeal).

## Discussion

This study reviewed and analyzed the results of 13 studies investigating the effect of drinking cessation on the risk of developing laryngeal and pharyngeal cancers. The results of the meta-analysis showed that there is a dose-response relationship between drinking cessation and a declining risk of laryngeal and pharyngeal cancers. Drinking cessation was associated with an average of 2% lower risk per year compared with current drinkers. This risk decline implies that, compared to the estimated risk of never drinkers, around 15% of the alcohol-related elevated risk of the laryngeal and pharyngeal cancers disappears after 5 years of drinking cessation. Moreover, the risk declined faster over first few years after drinking cessation. Hence, although a long time period is required for full risk reduction, there are substantial beneficial effects of alcohol cessation in the short term.

We found that it would take more than 35 years for the elevated risk of alcohol consumption to decrease to the level of never drinkers. Rehm et al. [8] showed that the risk of developing head and neck cancer among individuals who stopped drinking for more than 16 years was significantly higher than that among never drinkers (0.72 vs. 0.46). This long-lasting effect of drinking on the risk of disease has been documented for other types of cancers. For example, previous studies have reported that 16.5 (95% CI: 13–24) and 23 (95% CI: 14–70) years of abstention are required before the elevated risk of drinking disappears for oesophageal and liver cancer, respectively [29], [30]. There was no evidence of nonlinearity in the log-linear dose-response relations in our study. This implies that there was not enough evidence to assume that the risk of laryngeal and pharyngeal cancers initially increases after cessation and then decreases. However, as our model is log-linear, it implies that as time since cessation increases, the fall in the risk of developing laryngeal and pharyngeal cancers will decrease. This contradicts the findings of Rehm et al. [8], who reported an increase in the risk of head and neck cancer in the initial years after cessation. There are several possible explanations for this difference. First, the current study employed a different method; we used the generalized least squares model for trend estimation (GLST) of dose-response relationship, while Rehm et al. [8] used linear regression and cubic polynomial regression analysis. The GLST allows for effect modifiers to account for between-study heterogeneity, and accounts for the fact that all cessation groups have a common reference group [18]. It also allows estimation of the full time period until the alcohol-related elevated risk disappears completely (i.e. extrapolation). Second, separate analyses were conducted for laryngeal and pharyngeal cancers in the current study, while Rehm et al. [8] pooled these and other cancers as head and neck cancer. Finally, the current study included four more studies [7], [12], [27], [42] than Rehm et al. [8].

There was no evidence of heterogeneity in the studies of laryngeal cancer. However, there was considerable between-study heterogeneity in the studies of pharyngeal cancer. A significant part of this heterogeneity was explained by two studies, both of which were published before 2000 [27], [28]; one did not control for smoking [28], and the other did not use matching between cases and controls [27]. It has previously been shown that there is an interaction effect between smoking and alcohol consumption in two forms: a multiplicative risk increase [31], and a higher chance of quitting smoking after drinking cessation [32]. Hence, if a study does not control for smoking, the alcohol cessation coefficient will include the interaction effect and the slope is likely to be steeper compared to studies that control for smoking; our results showed that this was indeed the case. Regarding the increased risk after drinking cessation in the non-matched case-control study, there is no specific reason to believe that the risk should increase or decrease, and we think this is solely related to data in the study by Takezaki [27]. However, as between-study heterogeneity remained after controlling for these effect modifiers, a random-effects model was used to capture the unexplained heterogeneity.

As all included studies were non-experimental studies, control for potential confounders is important to estimate an unbiased effect of cessation on the risk. While, some of confounders such as age and smoking were controlled in the most studies, only two recent studies [7], [42] controlled for amount or length of drinking prior to cessation. It is therefore not obvious that if these studies estimated the unbiased average treatment effect of drinking cessation on the risk of laryngeal and pharyngeal cancers.

As we focused on occurrence of first cancer, the results of current study might not be applicable for the risk of recurrent cancer among people who already had one. For example, Day et al. [43] reported no risk reduction of second oral and pharyngeal cancers associated with quitting drinking at or after the diagnosis of first one. Estimating the effect of drinking cessation on the recurrence of laryngeal and pharyngeal cancers by a primary or secondary research study is a topic for future research.

The main strengths of the current study are the use of an appropriate method for capturing the dose-response relationship which abled us to estimate the OR per every additional year of cessation instead of estimating ORs for some time points after cessation (as was done by Rehm et al [8]), and the inclusion of the latest evidence on alcohol cessation and risk of laryngeal and pharyngeal cancers.

The results of the current study should be interpreted with some limitations in mind. First, the level, type, and length of consumption prior to quitting have been shown to have an impact on the risk of developing laryngeal and pharyngeal cancers [8], [33]–[36]. If these factors affect the risk decline after drinking cessation, then the results of the current study may be biased depending on the distribution of these factors among the former drinkers. On one hand, it is most likely that complete quitters are biased toward heavier consumption, which might mean that the current study’s estimate of the time taken for the alcohol-related elevated risk to completely disappear will be exaggerated for a light/moderate drinker. On the other hand, heavy consumers might have a faster risk decline due to their higher starting risk. The lack of data makes it impossible to pronounce on this issue, which is therefore left for future research. Second, all the identified studies were case-control studies; these are susceptible to recall, information and selection bias, which in turn may have affected the results of the current study. Third, there are some other potential confounders, such as body mass index and fruit and vegetable consumption, which could be related to both risk of cancer and drinking cessation. Data limitations hindered controlling for these confounders. Fourth, our estimated odds ratios for the risk of laryngeal and pharyngeal cancers for current drinkers compared with never drinkers was based solely on the studies identified for our meta-analysis, which is not representative of all studies comparing these two groups. This small number of studies resulted in a large confidence interval in our estimation, and hence the external validity of this specific estimation of the study is limited.

### Conclusions

Our results indicate that cessation of alcohol drinking is related to a reduction of the risk of developing laryngeal and pharyngeal cancers, though a substantial time period is required in order for the alcohol-related elevated risk to completely disappear. However, a substantial risk reduction can be seen in the short term (5–10 years), and drinking cessation should therefore be encouraged to reduce the incidence of laryngeal and pharyngeal cancers. Moreover, conducting large prospective studies in order to provide more reliable estimations are suggested. Moreover, estimating the effect of level and length of drinking before cessation on the risk of laryngeal and pharyngeal cancers is a question for future research.

## Supporting Information

Figure S1 **Dose-response relationship between years since quitting and relative risks of laryngeal cancer.** Lines with long dashes show the 95% confidence interval for the fitted nonlinear trend (solid line). Lines with short dashes show the linear trend.
(TIF)

Figure S2 **Dose-response relationship between years since quitting and relative risks of pharyngeal cancer.** Lines with long dashes show the 95% confidence interval for the fitted nonlinear trend (solid line). Lines with short dashes show the linear trend.
(TIF)

Figure S3 **Assessment of publication bias for studies of laryngeal cancer (graphs by time since quitting).**
(TIF)

Figure S4 **Assessment of publication bias for studies of pharyngeal cancer (graphs by time since quitting).**
(TIF)

Table S1 **Characteristics of studies of the risk of laryngeal and pharyngeal cancer following drinking cessation.**
(DOC)
