# Supplementary figures and images for: Alcohol Drinking Cessation and the Risk of Laryngeal and Pharyngeal Cancers: A Systematic Review and Meta-Analysis
Source: PLoS One. 2013 Mar 1;8(3):e58158. doi: 10.1371/journal.pone.0058158 (PMC3585880; doi:10.1371/journal.pone.0058158)

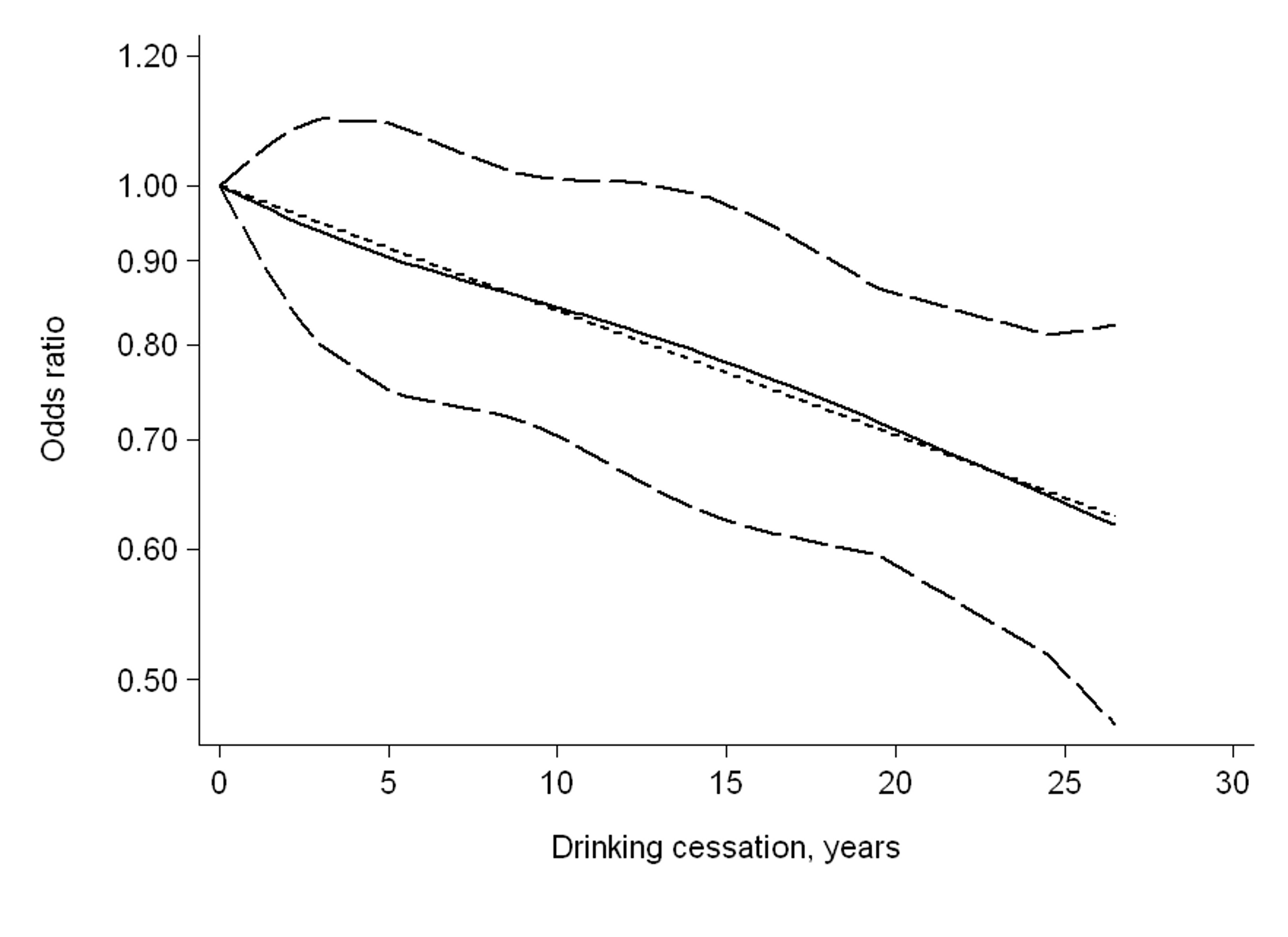

Supplement: Figure S1 — Dose-response relationship between years since quitting and relative risks of laryngeal cancer. Lines with long dashes show the 95% confidence interval for the fitted nonlinear trend (solid line). Lines with short dashes show the linear trend. (TIF) [file pone.0058158.s001.tif]

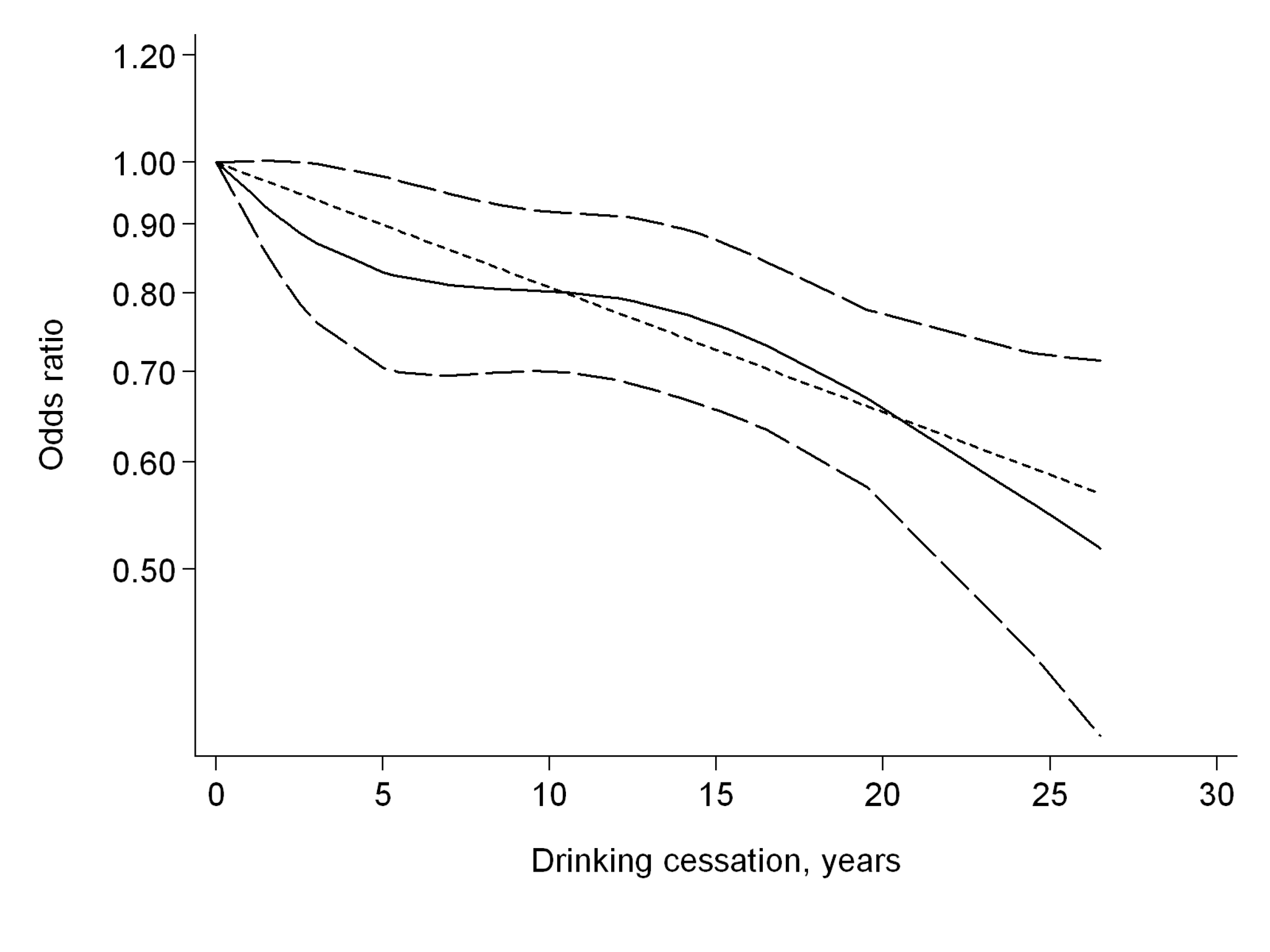

Supplement: Figure S2 — Dose-response relationship between years since quitting and relative risks of pharyngeal cancer. Lines with long dashes show the 95% confidence interval for the fitted nonlinear trend (solid line). Lines with short dashes show the linear trend. (TIF) [file pone.0058158.s002.tif]

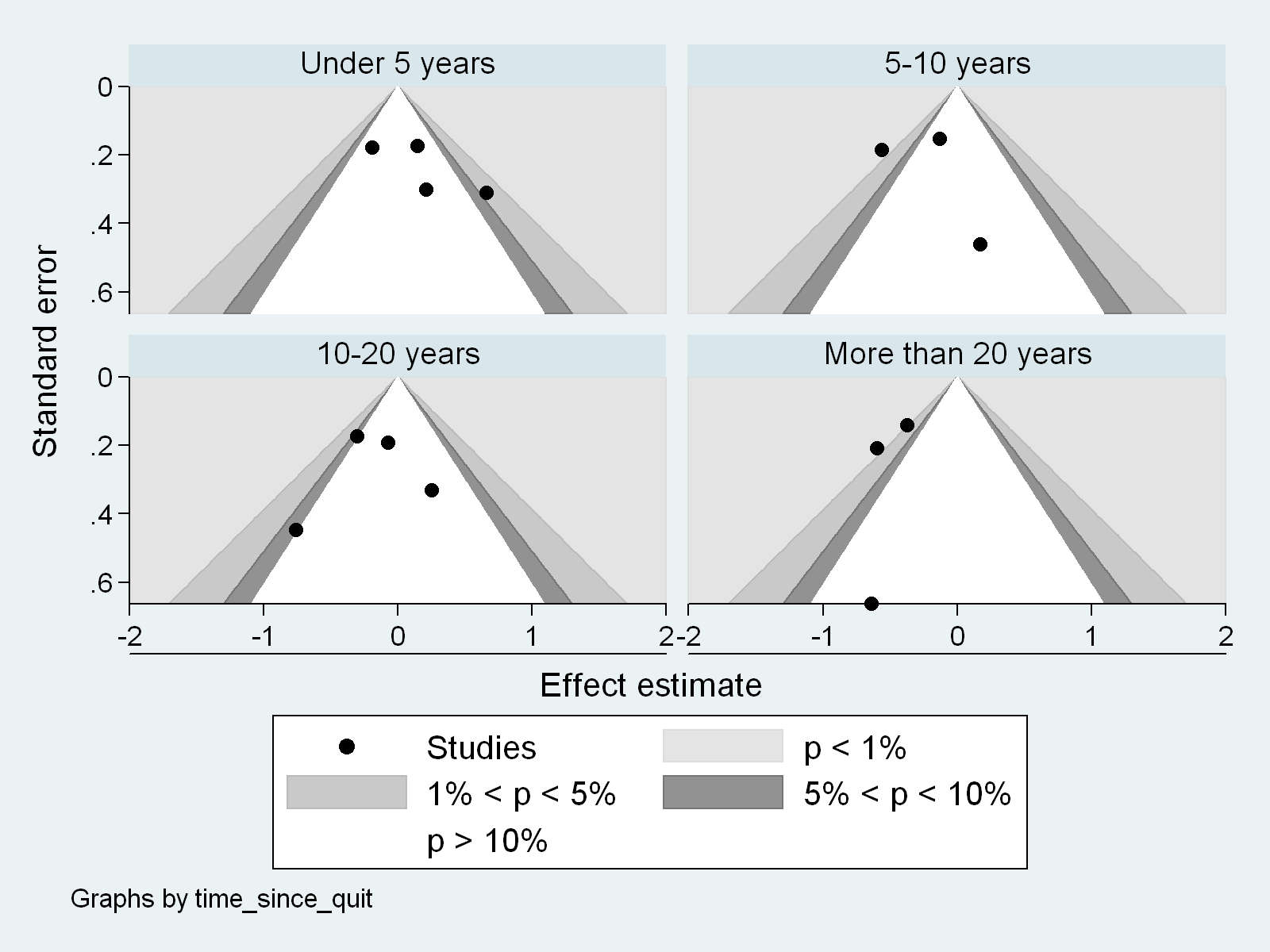

Supplement: Figure S3 — Assessment of publication bias for studies of laryngeal cancer (graphs by time since quitting). (TIF) [file pone.0058158.s003.tif]

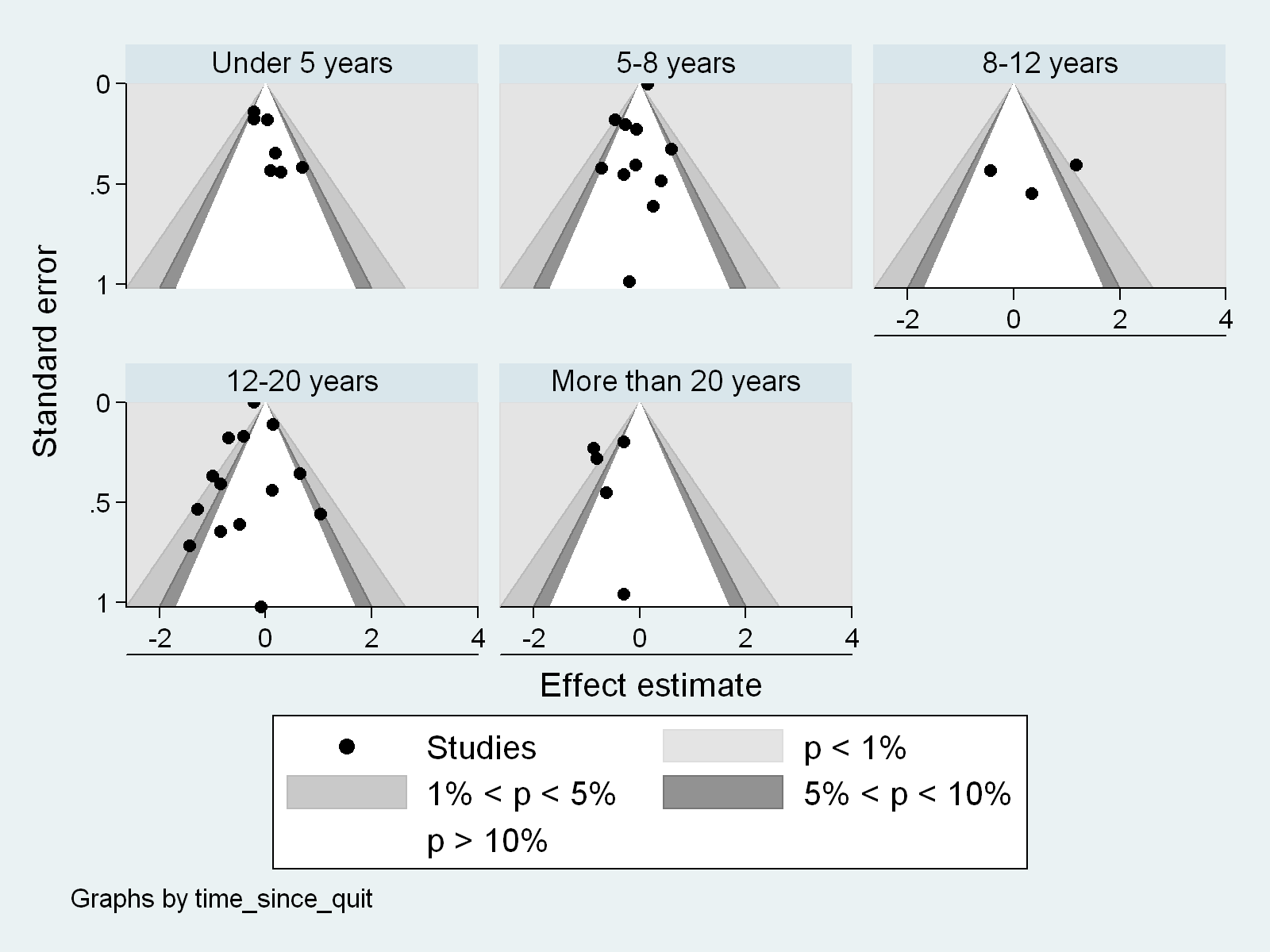

Supplement: Figure S4 — Assessment of publication bias for studies of pharyngeal cancer (graphs by time since quitting). (TIF) [file pone.0058158.s004.tif]
