# Supplementary material for: Alcohol Drinking Cessation and the Risk of Laryngeal and Pharyngeal Cancers: A Systematic Review and Meta-Analysis
Source: PLoS One. 2013 Mar 1;8(3):e58158. doi: 10.1371/journal.pone.0058158 (PMC3585880; doi:10.1371/journal.pone.0058158)
Supplement: Table S1 — Characteristics of studies of the risk of laryngeal and pharyngeal cancer following drinking cessation. (DOC) [file pone.0058158.s005.doc]

Table S1. Characteristics of studies of the risk of laryngeal and pharyngeal cancer following drinking cessation.

| Study | Histological confirmation of cases | Source of controls | Sample size (case/control) | Response rate case/control (%) | Matched case-control study (matching factors) | Controlling for potential confounders | Statistical analysis | Period until classified as a former drinker |
| --- | --- | --- | --- | --- | --- | --- | --- | --- |
| Altieri et al., 2002 | Yes | Hospital | 527/1297 | NA | Yes (A, G, C) | Yes (A, G, C, E, S) | Unconditional multiple logistic regression | 1 year |
| Castellsague et al., 2004 | Yes | Hospital | 375/375 | 76.5/91 | Yes (A, G) | Yes (A, G, C, E, S) | Unconditional multiple logistic regression | 1 year |
| De Stefani et al., 2004 | Yes | Hospital | 320/640 | 97.2/98.7 | Yes (A, R) | Yes (A, R, E, S, B, US) | Unconditional multiple logistic regression | 1 year |
| Franceschi et al., 2000 | Yes | Hospital | 754/1775 | NA | Yes (A, G) | Yes (A, G, C, E, S, I) | Unconditional multiple logistic regression | 1 year |
| Balaram et al., 2002 | NA | Hospital | 591/582 | NA/90 | Yes (A, G, C) | Yes (A, C, E, S, CH) | Unconditional multiple logistic regression | 1 year |
| Hayes et al., 1999 | Yes | Resident place and HCFA | 367/521 | 71/83 | Yes (A, G) | Yes (A, S) | Unconditional multiple logistic regression | 2 years |
| Garrote et al., 2001 | NA | Hospital | 200/200 | 88/79 | Yes (A, G) | Yes (A, G, R, E, S) | Unconditional multiple logistic regression | 1 year |
| Szymanska et al., 2011 | Yes | Hospital | 2026/1707 | 95/86 | Yes (A, G, C) | Yes (A, G, C, E, S, FC, AD) | Unconditional multiple logistic regression | 1 year |
| Takezaki et al., 1996 | Yes | Hospital | 266/36527 | 91.4 | No | Yes (A, G, S, YV) | Unconditional multiple logistic regression | 1 year |
| Takezaki et al., 2000 | Yes | Hospital | 62/11936 | 91.3 | No | Yes (A, S, YSV, RV) | Unconditional multiple logistic regression | 1 year |
| Martinez, 1969 | Yes | Hospital and community | 183/183 | NA | Yes (A, G, C, R) | No | X^2^ statistics | 0 year |
| Marron et al., 2009 | See included | See included | 9167/12593 | See included | See included | Yes ( A, G, RA, C, E, S, DF) | Random-effect logistic regression | 1 year |
| Rehm et al.,  2007 | See included | See included | - | See included | See included | See included | Linear and polynomial regression |  |

A: age, G: gender, S: smoking, E: education, YV: year of visit, YSV: year and season of visit, DF: drinking frequency, RA: race/ethnicity, C: centre, FC: fruit and cruciferous consumption, AD: alcohol gram per day, R: residence, CH: chewing habit, I: interviewer, US: urban/rural status, B: body mass index, RV: consumption of raw vegetables.
